# Machine learning-based prediction of low-value care for hospitalized patients

**DOI:** 10.1016/j.ibmed.2023.100115

**Published:** 2023-10-23

**Authors:** Andrew J. King, Lu Tang, Billie S. Davis, Sarah M. Preum, Leigh A. Bukowski, John Zimmerman, Jeremy M. Kahn

**Affiliations:** a Department of Critical Care Medicine, University of Pittsburgh School of Medicine, Pittsburgh, PA, USA; b Department of Biostatistics, University of Pittsburgh School of Public Health, Pittsburgh, PA, USA; c Department of Computer Science, Dartmouth College, Hanover, NH, USA; d Human-Computer Interaction Institute, Carnegie Mellon University School of Computer Science, Pittsburgh, PA, USA

**Keywords:** Evidence-based medicine, Resuscitation, Clinical decision support, Low-value care, Machine learning

## Abstract

**Objective:**

Low-value care (i.e., costly health care treatments that provide little or no benefit) is an ongoing problem in United States hospitals. Traditional strategies for reducing low-value care are only moderately successful. Informed by behavioral science principles, we sought to use machine learning to inform a targeted prompting system that suggests preferred alternative treatments at the point of care but before clinicians have made a decision.

**Methods:**

We used intravenous administration of albumin for fluid resuscitation in intensive care unit (ICU) patients as an exemplar of low-value care practice, identified using the electronic health record of a multi-hospital health system. We divided all ICU episodes into 4-h periods and defined a set of relevant clinical features at the period level. We then developed two machine learning models: a single-stage model that directly predicts if a patient will receive albumin in the next period; and a two-stage model that first predicts if any resuscitation fluid will be administered and then predicts albumin only among the patients with a high probability of fluid use.

**Results:**

We examined 87,489 ICU episodes divided into approximately 1.5 million 4-h periods. The area under the receiver operating characteristic curve was 0.86 for both prediction models. The positive predictive value was 0.21 (95% confidence interval: 0.20, 0.23) for the single-stage model and 0.22 (0.20, 0.23) for the two-stage model. Applying either model in a targeted prompting system could prevent 10% of albumin administrations, with an attending physician receiving one prompt every 4.2 days of ICU service.

**Conclusion:**

Prediction of low-value care is feasible and could enable a point-of-care, targeted prompting system that offers suggestions ahead of the moment of need before clinicians have already decided. A two-stage approach does not improve performance but does interject new levers for the calibration of such a system.

## 1. Introduction

De-implementing low-value care practices (i.e., costly medical practices that provide little or no benefit to patients) is a significant public health problem in the United States [1], costing up to $100 billion annually [2-4]. Low-value care harms patients by exposing them to unnecessary risks, consuming scarce resources, and overcomplicating care [5]. These issues are particularly prominent in hospitalized patients, where clinical decisions are made with extreme time pressure and high patient acuity creates a culture of doing more for patients [6].

To address the low-value care problem [7-9], implementation scientists have developed novel implementation science theories surrounding de-adoption [10] and have deployed dozens of different de-adoption strategies [11-13], such as interruptive alerts within the electronic health record (EHR) [14]. Unfortunately, the impact of interruptive alerts on reducing low-value care is modest [14-17].

A potential alternative to traditional interruptive alerts is a targeted prompting system that notifies clinicians earlier in the decision process, ideally immediately before a clinician realizes that a particular clinical decision needs to be made. Such a system would prospectively prime a high-valued decision rather than retrospectively correct a low-valued one [18]. A critical barrier preventing the deployment of a targeted prompting system is identifying when a notification ought to be generated. We aim to address this barrier by developing a prediction model that identifies situations with a high likelihood that a clinician will order a low-value practice.

## 2. Methods

### 2.1. Low-value care of focus

As a demonstrative example of low-value care, we focus this study on administering IV albumin for fluid resuscitation in the intensive care unit (ICU). The relative merits of IV albumin versus alternative fluid choices have been extensively reviewed elsewhere [19-22]. Briefly, IV fluid resuscitation is a mainstay of critical care and is indicated whenever patients are experiencing end-organ hypoperfusion due to intravascular depletion. Most typically, this occurs in the setting of sepsis or other types of relative hypovolemia. By providing fluid resuscitation, the aim is to maintain organ perfusion (i.e., blood flow) and substrate (e.g., oxygen) delivery while other more definitive treatments (e.g., antibiotics) are taking effect [23].

Once the decision is made to administer IV fluid, clinicians have several options for the type of fluid. One major category of fluid is electrolytes dissolved in sterile water. This category is called crystalloid, and common types include normal saline or Ringer’s lactate. A second major category is large proteins or starches sustained in sterile water. This category is called colloid; the most common type is human albumin. In theory, IV albumin should be a superior resuscitation fluid to crystalloid since its large molecular structure should stay in the intravascular space, preventing complications from extravascular spillover such as pulmonary edema. However, in practice, IV albumin causes just as many complications as other fluids. In head-to-head randomized trials, albumin is consistently associated with similar or worse outcomes than crystalloid [24,25]. At the same time, albumin is exponentially more costly than crystalloid solutions, making it a classic example of low-value care in hospitalized patients [26,27].

### 2.2. Conceptual model

**Fig. 1.**
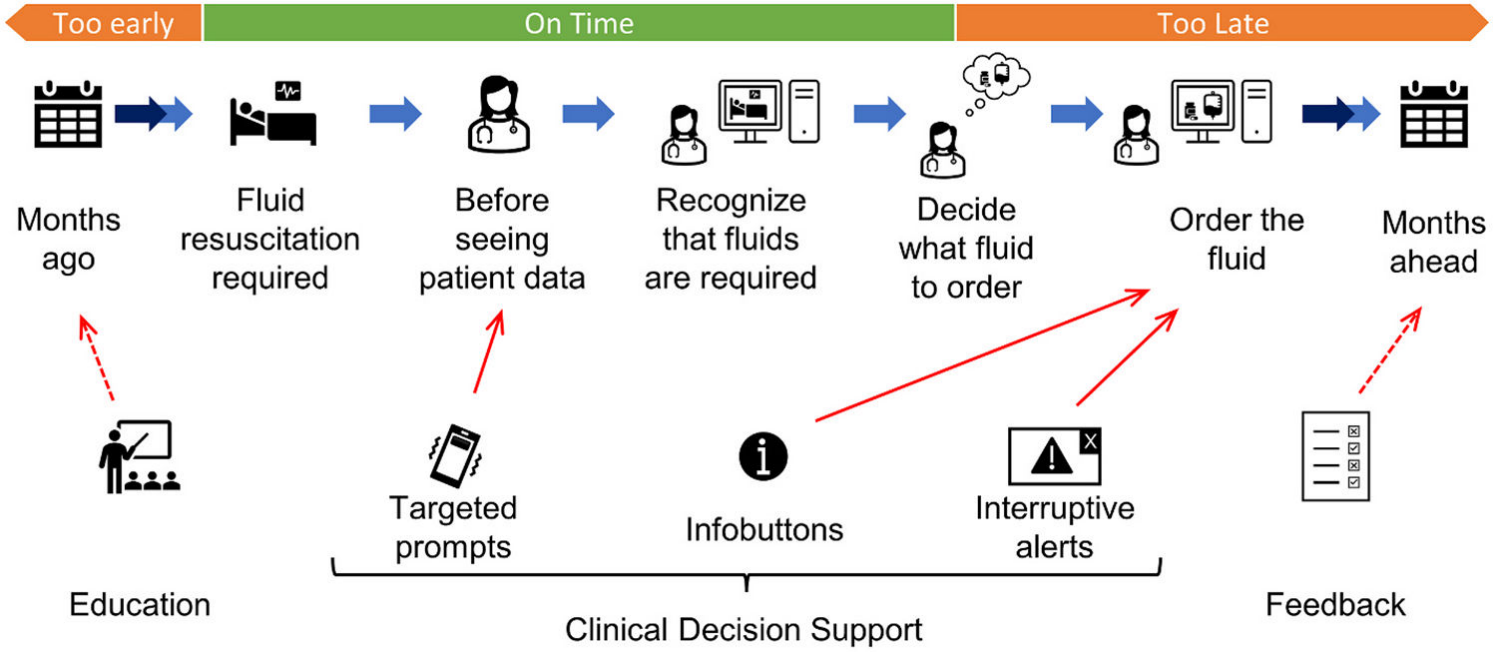
Strategies for reducing the use of albumin-containing fluids for resuscitation. Education is too early and may be forgotten. Infobuttons and interruptive alerts are too late because it is difficult to change a clinician’s mind after they have already decided what fluid to order. Targeted prompts might work because they will provide information right before the clinician recognizes that they need to order fluids.

Hospital leaders have numerous strategies for guiding clinicians toward higher-value treatment decisions (Fig. 1) [11]. Most of these strategies intervene too late to impact the decisions made for an individual patient. By design, *feedback* about past decisions can only improve decisions for future patients. Clinical decision support-based strategies, such as *infobuttons* (that show a hover box of relevant information [28]) and *interruptive alerts* (that trigger a pop-up window prompting a specific action), are usually on EHR order entry screens, which are often not viewed by clinicians until after they decide what to order.

A potential alternative strategy for higher-value decisions is to prompt^1^ clinicians for a particular evidence-based practice immediately before the moment of need, i.e., before they decide on a particular treatment course. As with other clinical decision support systems, prompts must be targeted so clinicians are not overwhelmed by irrelevant information [29]. Therefore, accurate models for predicting treatment decisions are needed.

### 2.3. Study design and setting

We performed a comparative machine learning study using retrospective EHR data from critical care patients discharged from UPMC hospitals between January 1st, 2018 and September 30th, 2020. UPMC is a multi-hospital integrated health system in the mid-Atlantic region of the United States. The data covers 38 ICUs in 18 hospitals and includes 87,489 hospitalizations requiring intensive care. Research use of this limited dataset was approved by the University of Pittsburgh Human Research Protection Office (IRB #19040420).

### 2.4. Data processing

ICU admissions were identified from location codes in the data. Hospitalizations not requiring ICU admission were excluded from the sample. Subsequently, each eligible hospitalization was windowed into fixed, 4-h periods: 07:00–10:59, 11:00–14:59, 15:00–18:59, 19:00–22:59, 23:00–02:59, and 03:00–06:59. A 4-h period length was chosen because we hypothesized it was long enough to include sufficient data for prediction while also short enough to provide meaningful time for clinical decision making. Missing values were not imputed or carried over from prior periods.

Within each period, we defined approximately 400 independent variables derived from EHR data from the current period or prior periods (e.g., highest blood pressure, urine output), as well as time-invariant variables that did not change across periods (e.g., patient age, patient gender). Clinicians with experience in the direct care of patients with critical illness guided the selection of broad domains and specific variables within those domains. Domains included patient characteristics, vital signs, laboratory test results, medication administration, and procedures [30]. A list of the variables is provided in Appendix A.

For each 4-h period, we also assigned two dependent variables: (a) a binary variable specifying whether IV albumin was administered in the subsequent period, and (b) a binary variable specifying whether any resuscitation fluid was administered in the subsequent period, which we defined as ≥ 250 ml fluids given during the period. These dependent variables are defined based on data from the immediately following period because our goal was to predict the actions (i.e., orders) that will occur in the immediate future.

Periods within a hospitalization that occurred after the first administration of IV albumin were excluded because it is not valuable to predict the administration of IV albumin after it has already begun. After data processing, all periods were randomly split 50/50 into training and testing sets, each treated independently.

### 2.5. Modeling approach

Our overarching goal was to develop a model that predicts albumin administration before a clinician decides to place the order. The standard approach for building a supervised machine learning model is to make the prediction in a single step. That is, the input is the vectorized set of independent variables, and the output is an estimate of the dependent variable. However, because albumin administration is a relatively rare event, a single-step prediction model might have an unacceptably high false positive rate [31,32]. Additionally, we assume that clinicians will be more annoyed by irrelevant prompts (i.e., an albumin prompt when none of their patients require fluid resuscitation) than they will be by relevant prompts (i.e., an albumin prompt when at least one of their patients requires or will soon require fluid resuscitation), even if they were not going to order the low-value practice (i.e., an albumin containing fluid). Under this framing, it is helpful in not just predicting when a low-value practice will be ordered but also when there is a situation where alternatives to the low-value practice will be ordered (i.e., any fluid for resuscitation).

To capture the two-step nuance of predicting both (i) when a patient will receive any resuscitation fluid and (ii) when a patient will receive a specific resuscitation fluid, we propose a two-stage machine learning model. For this study, the first stage of the two-stage model predicts which patients will receive any resuscitation fluid (as defined by receiving ≥250 ml of intravenous fluids) or an albumin-containing fluid within the subsequent 4-h period. Then, among this population of patients predicted to receive any resuscitation fluids, predict which of those orders will be for an albumin-containing fluid.

**Fig. 2.**
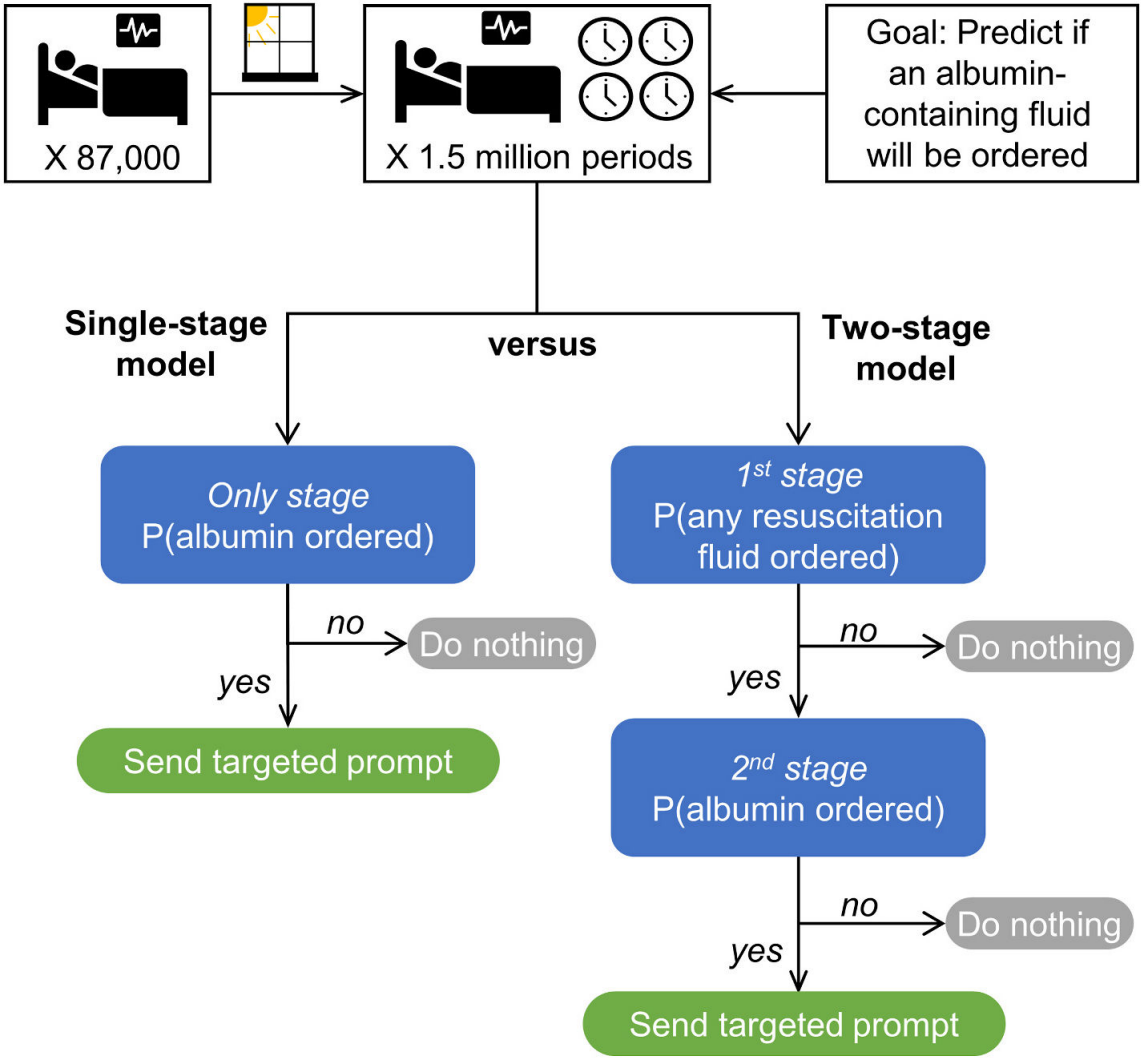
Experimental setup comparing the performance of single-stage and two-stage models with the goal of anticipating orders of an albumin-containing fluid. The 2nd stage of the two-stage model is applied to a patient population that is enriched for those patients who will receive an order for any resuscitation fluid.

To test the proposed approach, we trained a single-stage and a two-stage model on the same data and compared their performance when predicting the administration of an albumin-containing fluid. An overview of this experimental setup is shown in Fig. 2.

We used five-fold cross-validation on the training set for model fitting, hyperparameter tuning, and selection. Each model used extreme gradient boosting learning, which was implemented by the caret package (version 6.0–93) in R (version 4.2.0). We chose extreme gradient boosting for prediction because it has been shown to have state-of-the-art performance on high-dimensional ICU data sets [33].

### 2.6. Model evaluation

We assessed the final performance of each model on the testing set. We report the receiver operating characteristic (ROC) curve, the area under the curve (AUC), and the confusion matrix for each model and stage.

To make the single-stage and two-stage model results more directly comparable, we repeated the experiment while setting the prediction threshold for the only stage of the single-stage model and the second stage of the two-stage model to be the values that resulted in a 10% sensitivity on the training set. (Because the first stage of the two-stage model is an intermediate prediction task, we chose its prediction threshold as the value that maximized F1 score on the training set). By choosing equivalent model sensitivities, the positive predictive values of the two models are directly comparable. Positive predictive value is the metric of greatest interest because the targeted prompting system “pushes” information to clinicians. When clinicians receive information they did not request, it is critical that the information is a true positive as often as possible. Otherwise, the clinicians might quickly become annoyed. This approach contrasts with other situations (i.e., laboratory testing) where information is “pulled” (i.e., requested) by the clinician. In the latter case, clinicians have already committed to seeing the information when they place the order. Thus, annoyance becomes less of a risk, and sensitivity or specificity are the most important metrics. We reported testing set performance as positive predictive value (TP/(TP + FP)), sensitivity (TP/(TP + FN)), specificity (TN/(TN + FP)), negative predictive value (TN/(FN + TN)), and F1 score (TP/(TP+0.5(FP + FN))), where TP, FP, FN, and TN correspond to the four confusion matrix outcomes: true positive, false positive, false negative, and true negative. Bootstrap resampling is performed 1000 times on the testing set to estimate 95% confidence intervals.

Additionally, we calculated the number of alerts an ICU physician would be expected to receive each day. To calculate alerts per day, we first calculated the alert rate (number of alerts per 4-h period) using the following equation: (TP + FP)/(TP + FP + FN + TN). To calculate alerts per ICU-day, we next multiplied alerts per period times periods per patient-day (6 for a 4-h period) and times patient-days per ICU-day (12 for a 12-patient unit). Finally, we calculated the inverse of alerts per ICU-day to determine how many days are between each alert.

Lastly, we conducted feature importance experiments for each model and stage using Shapley additive explanations to determine if the different modeling approaches led to differences in the top ten selected features [34]. These top ten features are shown in a Venn diagram with one circle for each model.

## 3. Results

The final processed data set included 87,489 inpatient hospitalizations and 1,440,710 periods. Patient characteristics are shown in Table 1, and period characteristics in the training and testing sets are shown in Table 2. The dependent variables, administration of albumin in the subsequent period and administration of any resuscitation fluid in the subsequent period, were True in 9844 (0.68%) and 251,836 (17.48%) of the periods, respectively.

**Fig. 3.**
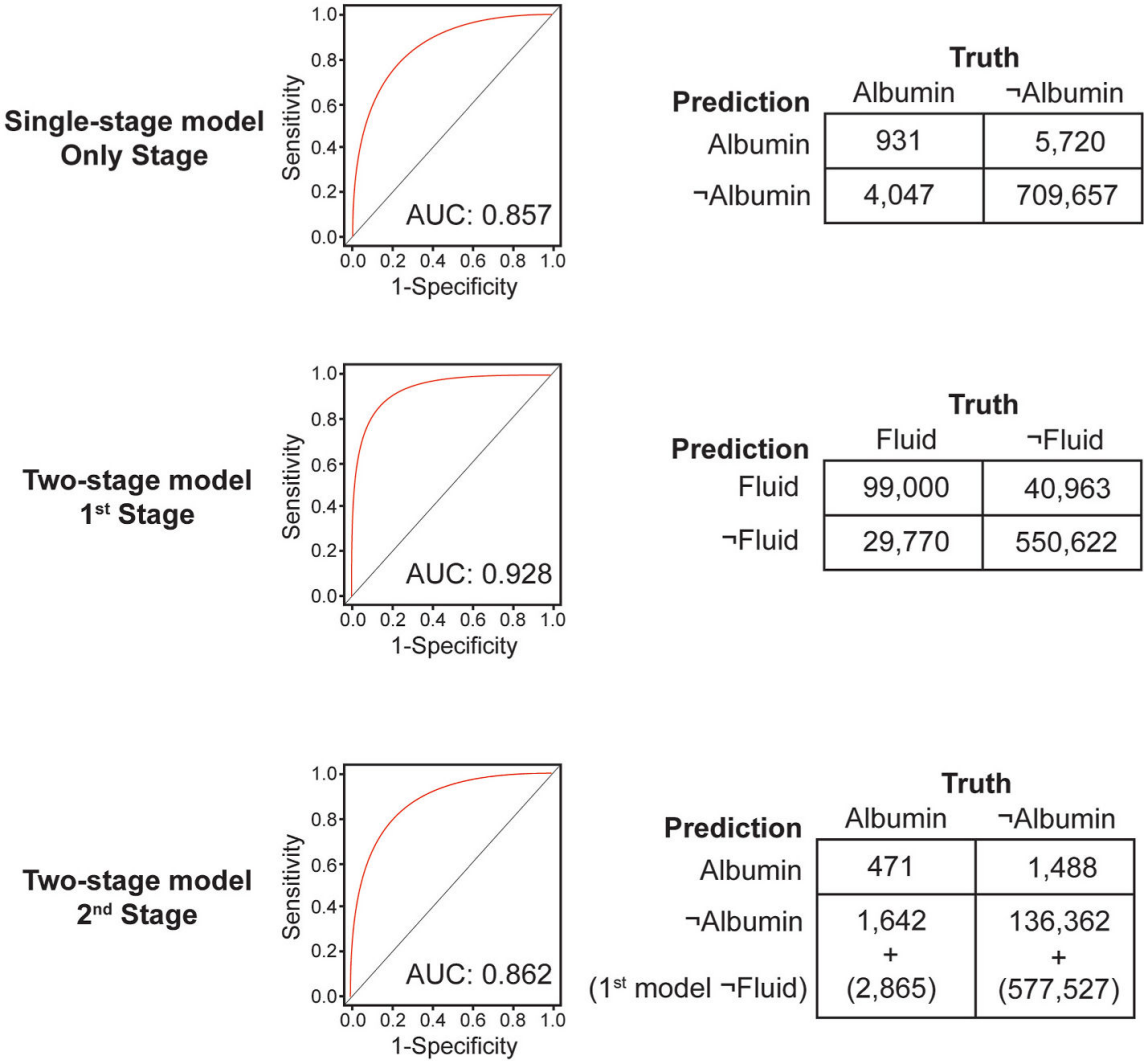
Results of the single and two-stage models. The 2nd stage of the two-stage model is only applied to periods that were predicted to receive fluid by the 1st stage of the two-stage model. Therefore, the ROC curve of the 2nd model does not include the periods that were predicted not to receive an order for a resuscitation fluid. For a fair comparison with the single-stage model, the confusion matrix has the periods predicted not to receive fluid added back in.

ROC, AUC, and confusion matrixes for all model stages on the testing set are shown in Fig. 3. The positive predictive values of the two models when the prediction threshold was selected for a 10% sensitivity are shown in Table 3. From these numbers, we calculated the alert rate to be 0.003314 alerts/period, equating to 1 alert for every 4.2 ICU-days.

**Fig. 4.**
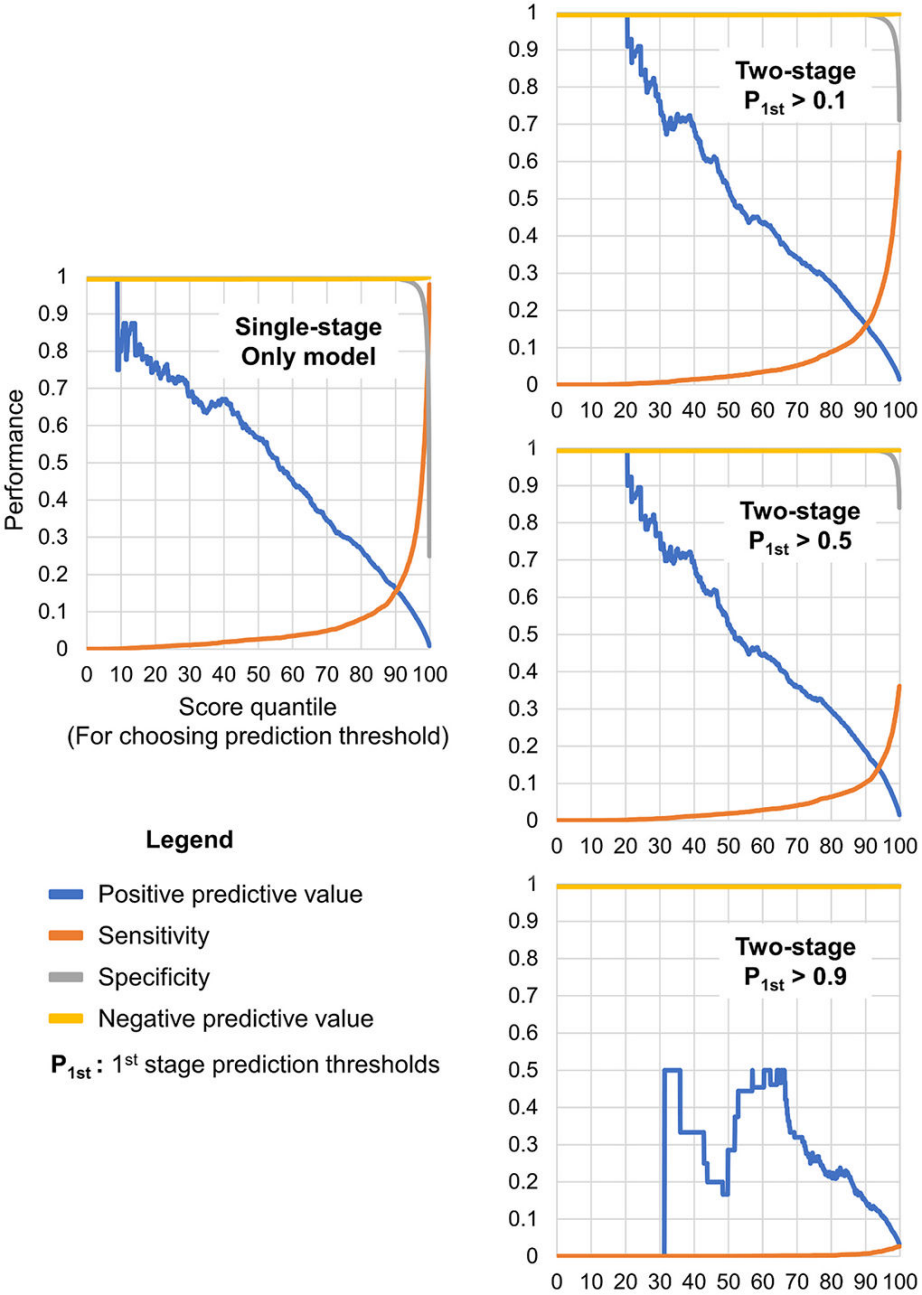
Visualization of performance at different prediction thresholds. The single-stage model has a single point of threshold calibration, whereas the two-stage model has two points of threshold calibration.

**Fig. 5.**
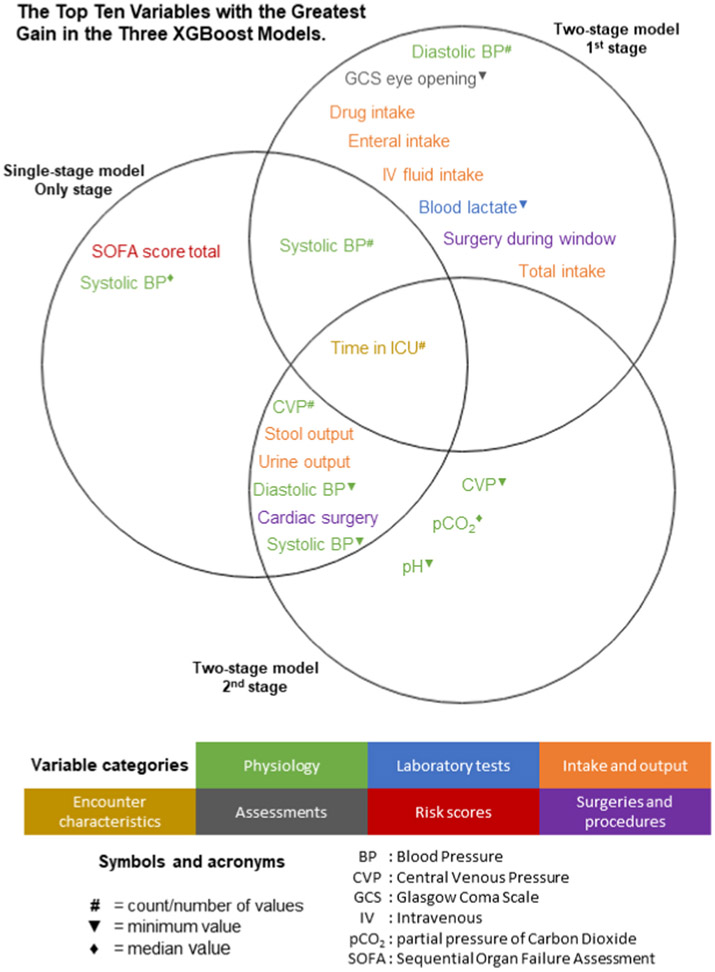
A Venn diagram of the top ten most important features (as found using Shapley additive explanations) for all model stages.

Fig. 4 depicts classification accuracy across different prediction thresholds. The two-stage model has a threshold for both model stages, allowing greater calibration flexibility depending on local needs and the low-value care of interest. Fig. 5 shows how the feature importance varied between the different model stages (see Appendix B for the original Shapley importance figures). As expected, the features used by the only stage of the single-stage model and the second stage of the two-stage model are most similar because these models perform the same task: predicting the administration of an albumin-containing fluid.

## 4. Discussion

We used two modeling approaches to predict when a patient would receive albumin for fluid resuscitation. This clinical prediction task is difficult because administration of albumin is rare (<7 out of every 1000 periods in our dataset), and, as we infer from our results, the patient’s clinical state is a weak predictor of the use of IV albumin. Supporting this conclusion, we found predicting the administration of resuscitation fluid (first stage of the two-stage model) to be higher performing than predicting the administration of IV albumin (only stage of the single-stage model and second stage of the two-stage model). We hypothesized that our novel two-stage model would lead to greater positive predictive value because the first stage of the model enriches a subpopulation with a high likelihood of receiving resuscitation fluid. However, this approach led to overall results that were not significantly better than the single-stage model. Despite these results, a two-stage model may still be appropriate for some use cases as it provides additional calibration and model interpretation levers.

A key innovation of this work is using machine learning to intervene in the use of evidence-based practice rather than predicting a clinical outcome or making a diagnosis [35]. Most prediction-focused machine learning applications in hospital medicine are used to predict clinical outcomes such as mortality [36-38] or whether the patient has a specific clinical condition like sepsis [39-41]. A fundamental limitation of these approaches is that delivering this information back to clinicians is unlikely to change practice. In the case of mortality prediction, there is little that the clinician can do to affect mortality based on the prediction, i. e., no specific levers for practice change are provided by telling a clinician a patient is at high risk of death. In the case of sepsis prediction, clinicians are often already aware of the diagnosis, and so telling the clinician that the patient has sepsis is unlikely to change the patient’s care trajectory, as has been borne out in clinical studies [42,43].

In contrast, our study is one of the first to use machine learning to predict the use of evidence-based care practices. This is an important distinction because such models can enable targeted prompting systems that preemptively guide clinicians towards adopting evidence-based practices rather than interrupting a clinician’s workflow and attempting to change a decision after it has been made. As a result, the system can also optimize its prompts according to the needs of all a clinician’s patients rather than focusing on one patient at a time. For example, if a clinician is going to make a fluid resuscitation decision for two or more patients on the same day, a single targeted prompt of the preferred resuscitation fluids would likely benefit all the patients rather than burdening the clinician with a prompt for each of the qualified patients. Optimizing across all a clinician’s patients becomes even more beneficial as the system’s scope grows to include dozens of different evidence-based practices.

Another distinction of predicting treatment decisions rather than patient outcomes is that intervening in the former is more likely to train a clinician towards habitually adopting evidence-based practices. This is the chronologic advantage of influencing clinician actions: prompts presented today can reduce the prevalence of a low-value care in the future because treatment decisions are repeatable. Therefore, if a system can influence clinicians, even if only a few clinicians per year, into habitually making higher value decisions, benefits compound. It also means that a relatively low model sensitivity may be acceptable because it is essential that the clinicians continue to receive prompts over time. If a clinical decision support system is more annoying than helpful, clinicians will ignore or mute it, reducing the system’s sensitivity to zero.

### 4.1. Limitations

This work has a few limitations to consider.

1. The experiments used retrospective EHR data from a single health system; however, these data did include nearly one hundred thousand ICU admissions from 18 hospitals and 38 ICUs. The units included rage from highly specialized ICUs in quaternary care hospitals to ICUs in community hospitals without 24-hr intensivist staffing. The patients included reflect that of the ICU population of the geographic region given that we only excluded patients if their ICU stay was less than 24 h.
2. During data processing, we used 4-h periods without data carryover or imputation to represent a patient’s clinical state. Different period sizes, predictor variables, or implementation strategies could change our results.
3. Our low-value care of focus (albumin for fluid resuscitation) is an example with clearly recommended alternatives. We have yet to consider how a targeted prompting system may function differently for other evidence-based practices without clearly recommended alternatives such as ventilator management decisions [44].

### 4.2. Future work

Moving forward, we have three immediate next steps. (i) Determine the performance of the model with the inclusion of clinician characteristics. We believe that knowledge of an individual clinician’s past treatment patterns, especially those regarding albumin administration, will be helpful when predicting future orders of albumin and may enable “customized” prompts tailored to each specific clinician. (ii) Determine the prospective performance of the model. Before a model could be deployed in a clinical trial, various adoptions must be made for it to run on live clinical data. We will perform these adaptations and evaluate model performance on active patient cases. (iii) Optimize the user experience of using a targeted prompting system [45]. We will work with clinicians to realize the desired changes in clinical practice.

## 5. Conclusions

Prediction of treatment decisions is an important next step in increasing the adoption of evidence-based practices. Even moderately performing models might support a targeted prompting system that is better accepted by clinicians than the interruptive alerting systems currently used and result in compounding improvements in evidence adoption. Future work will evaluate if these models benefit from including clinician characteristics or identities as features, if the results are reproducible on real-time prospective data, and if the targeted prompting system is ready for a clinical trial.

## Supplementary Material

1

## Figures and Tables

**Table 1:** Patient characteristics.

	No. (%)
**Demographic information**	
Total No	87,492^a^
Age, median (IQR), y	65 (54–76)
Sex	
Female	40,678 (46.5%)
Male	46,814 (53.5%)
Race	
Black	9737 (11.1%)
White	72,414 (82.8%)
Other/multiracial/unspecified	5341 (6.1%)
Died during hospitalization	
No	77,263 (88.3%)
Yes	10,229 (11.7%)
**Comorbidities** ^b^	
Total No.	87,490
Acquired immune deficiency syndrome	147 (0.2%)
Alcohol abuse	7131 (8.2%)
Deficiency anemia	19,870 (22.7%)
Rheumatoid arthritis/collagen	3577 (4.1%)
Blood loss anemia	1070 (1.2%)
Congestive heart failure	25,061 (28.6%)
Chronic pulmonary disease	29,973 (34.3%)
Coagulopathy	12,208 (14.0%)
Depression	19,911 (22.8%)
Diabetes without chronic complications	9677 (11.1%)
Diabetes with chronic complications	21,457 (24.5%)
Drug abuse	3987 (4.6%)
Hypertension	63,041 (72.1%)
uncomplicated	31,706 (36.2%)
complicated	31,335 (35.8%)
Hypothyroidism	15,462 (17.7%)
Liver disease	8522 (9.7%)
Lymphoma	1217 (1.4%)
Fluid and electrolyte disorders	42,908 (49.0%)
Metastatic cancer	4829 (5.5%)
Other neurological disorders	18,384 (21.0%)
Obesity	20,139 (23.0%)
Paralysis	7962 (9.1%)
Peripheral vascular disorders	11,359 (13.0%)
Psychoses	6243 (7.1%)
Pulmonary circulation disorders	3922 (4.5%)
Renal failure	19,391 (22.2%)
Solid tumor without metastasis	5593 (6.4%)
Peptic ulcer disease excluding bleeding	2278 (2.6%)
Valvular disease	14,515 (16.6%)
Weight loss	16,498 (18.9%)
Elixhauser Comorbidity Index, median (IQR) [range]	5 (3–6) [0–17]
**Windowing of temporal EHR data into 4-hr periods**	
Periods per patient in analysis, median (IQR)	11 (6–20)
Total No. of periods	1,440,710
SOFA, median (IQR) [range]	3 (1–5) [0–24]
Receiving Invasive Mechanical Ventilation	
No	925,623 (64.2%)
yes	515,087 (35.8%)

Abbreviations: BMI, body mass index (calculated as weight in kilograms divided by height in meters squared); IQR, interquartile range; SOFA, sequential organ failure assessment.

a Individual patients are counted more than once if they had more than one hospitalization requiring intensive care.

b Comorbidity definitions are based on Elixhauser Comorbidity Index V2020.1.

**Table 2:** Four-hour period characteristics.

	Training set	Testing set
Number of periods	720,355	720,355
Resuscitation fluid orders (excluding albumin), N	124,633	123,792
Albumin-containing fluid orders, N	4866	4978

**Table 3:** Performance with 95% confidence interval at 10% sensitivity.

	Single-Stage	Two-Stage^a^
Positive predictive value	0.214 (0.197, 0.231)	0.217 (0.201, 0.233)
Sensitivity	0.106 (0.098, 0.115)	0.104 (0.096, 0.113)
Specificity	0.997 (0.997, 0.997)	0.997 (0.997, 0.998)
Negative predictive value	0.994 (0.994, 0.994)	0.994 (0.994, 0.994)
F1 score	0.142 (0.131, 0.153)	0.141 (0.130, 0.151)

a 1st stage cutoff was chosen to maximize F1 score.

## Data Availability

The data underlying this article cannot be shared publicly due to institutional policies that protect the privacy of individuals whose data were used in the study.

## Statement of significance

- Problem or Issue: Low-value care unnecessarily increases health care costs and can harm patients.
- What is Already Known: Existing strategies including decision support tools are only minimally effective at reducing low-value care.
- What this Paper Adds: A machine learning-based prediction model for low-value care is accurate and would enable a decision support system that provides suggestions for high-value alternatives prior to rather than after the time of decision. Single-stage and two-stage prediction models are comparably accurate but may differ in application in a live system.

## Footnotes

1 Here and throughout the paper, prompt is intended to encompass any means of electronically informing a clinician (consciously or subconsciously), for example, pop-up alerts, text-based reminders, or guiding choices through EHR defaults.

## Abbreviations

AUC: Area under the receiver operating characteristic curve
HER: electronic health record
FN: false negative
FP: false positive
ICU: intensive care unit
IV: intravenous
ROC: receiver operating characteristic
TN: true negative
TP: true positive
