# Supplementary material for: Machine learning-based prediction of low-value care for hospitalized patients
Source: Intell Based Med. Author manuscript; Available in PMC 2023 Dec 21. (PMC10735238; doi:10.1016/j.ibmed.2023.100115)

**Supplemental Material**

**Appendix A: Independent variables representing a four-hour window.**

- Age
- Sex
- Height
- Weight
- Race and ethnicity
- Number of 4-hour windows since hospital admission (i.e., Time in the hospital)
- Number of 4-hour windows since ICU admission (i.e., Time in the ICU)
- Code status
- Surgery and procedures (one-hot encoding of common codes)
- Vasopressor administration (current and prior)
- Delirium scores (CAM-ICU)
- Sequential Organ Failure Assessment (SOFA) score
- Respiratory support (type and settings)
- Input and output (total and nset for different categories)
- Medication administration (one-hot encoding of 200 common drug-route combinations)
- Mean, median, minimum, maximum, count, and most recent value of each of the following measurements: bicarbonate, bilirubin, blood urea nitrogen (BUN), cyclic citrullinated peptide (CCP) antibodies, creatinine, central venous pressure (CVP), diastolic blood pressure, glucose, heart rate, intracranial pressure, international normalised ratio (INR), lactate, partial pressure of oxygen (PaO2), partial pressure of carbon dioxide (PCO2) blood pH, platelets, respiratory rate, oxygen saturation of arterial blood (SaO2), sodium, oxygen saturation by pulse oximetry (SpO2), systolic blood pressure, temperature.
- Minimum of the following: Glasgow Coma Scale (GCS) eye, GCS motor, GCS verbal.

**Appendix B: Independent variables representing a four-hour window.**

**Supp Fig 1:** Shapley importance figure for two-stage model 1^st^ stage


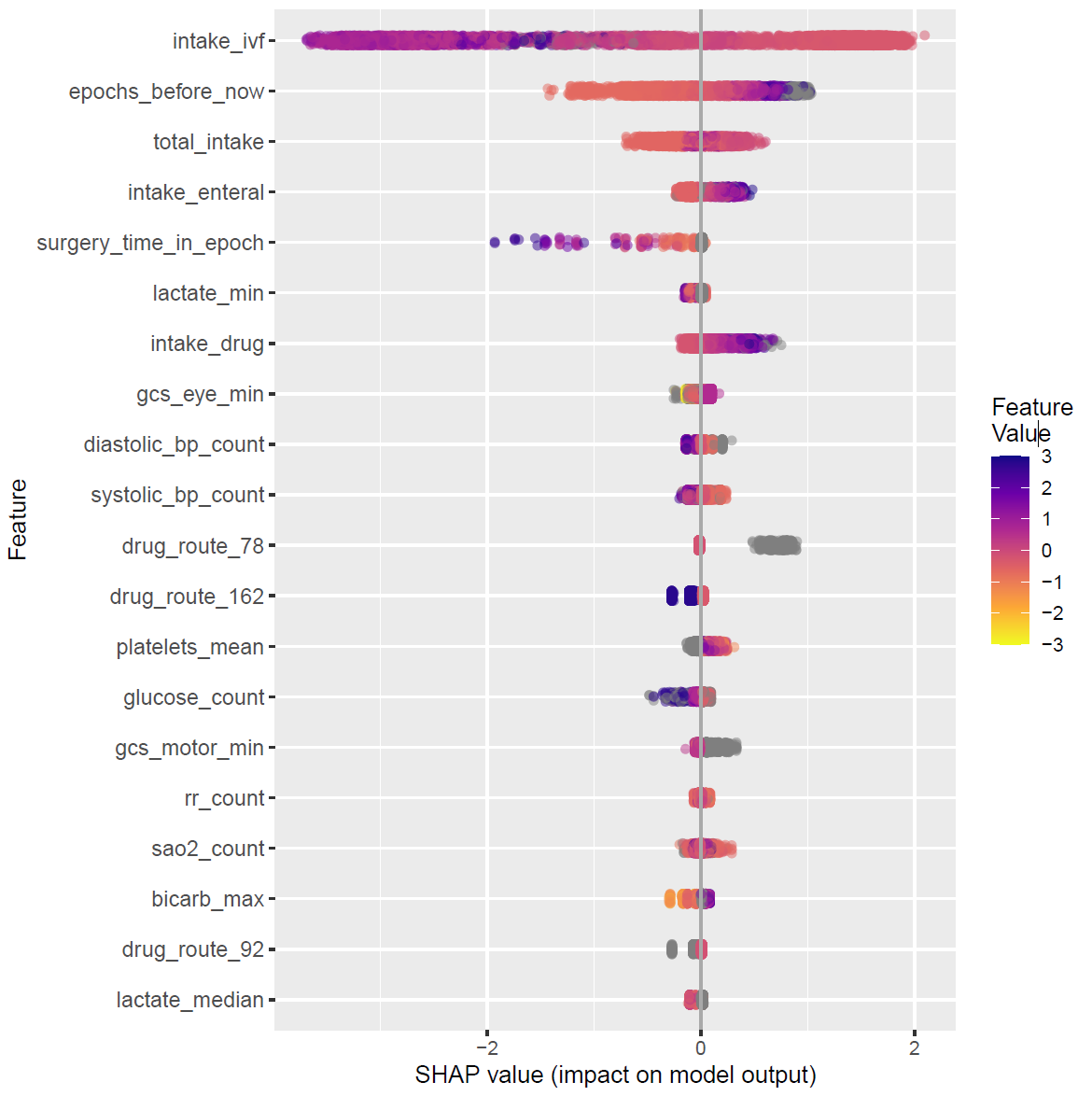


**Supp Fig 2:** Shapley importance figure for two-stage model 2^nd^ stage


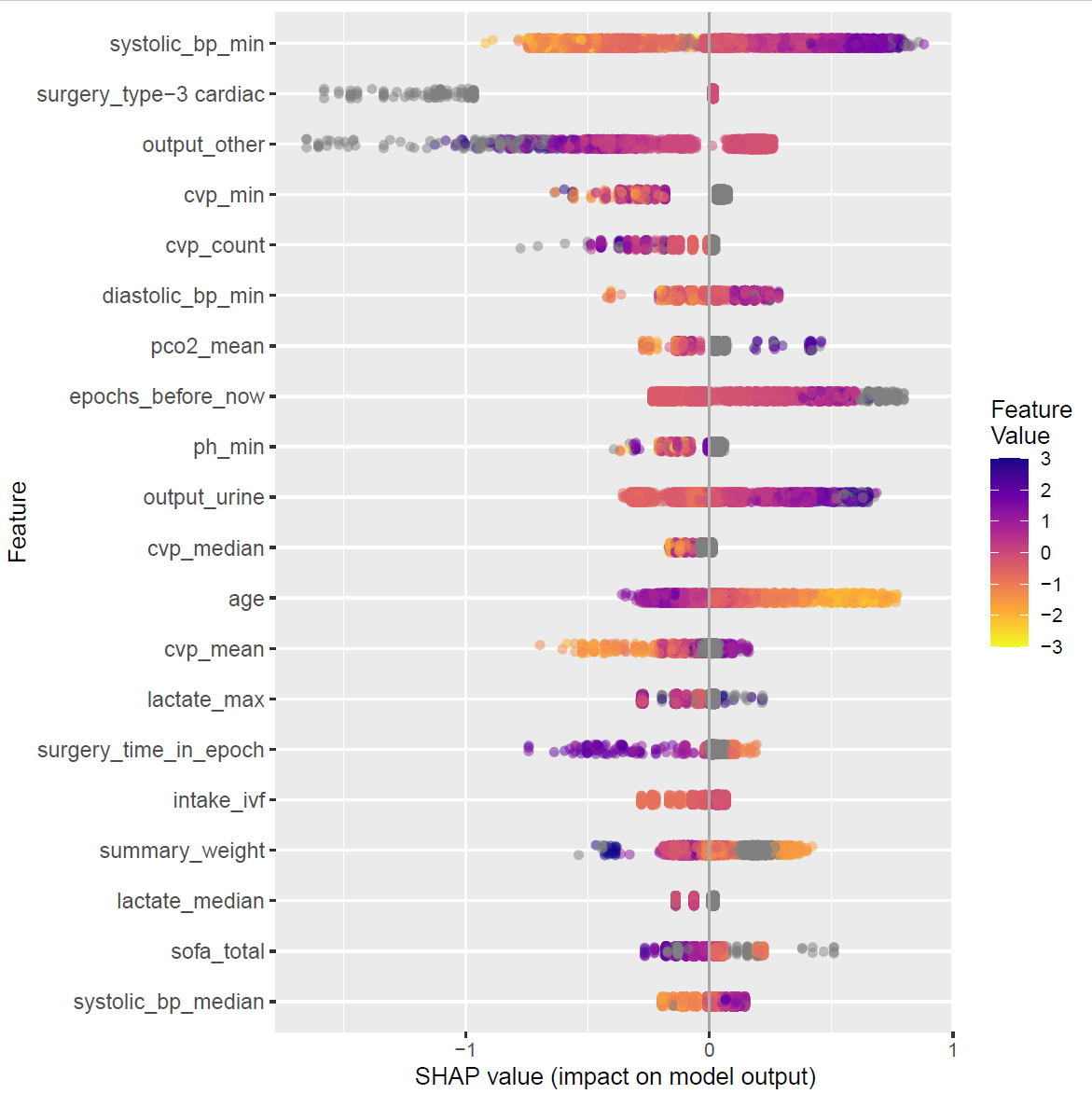


**Supp Fig 3:** Shapley importance figure for single-stage model only stage


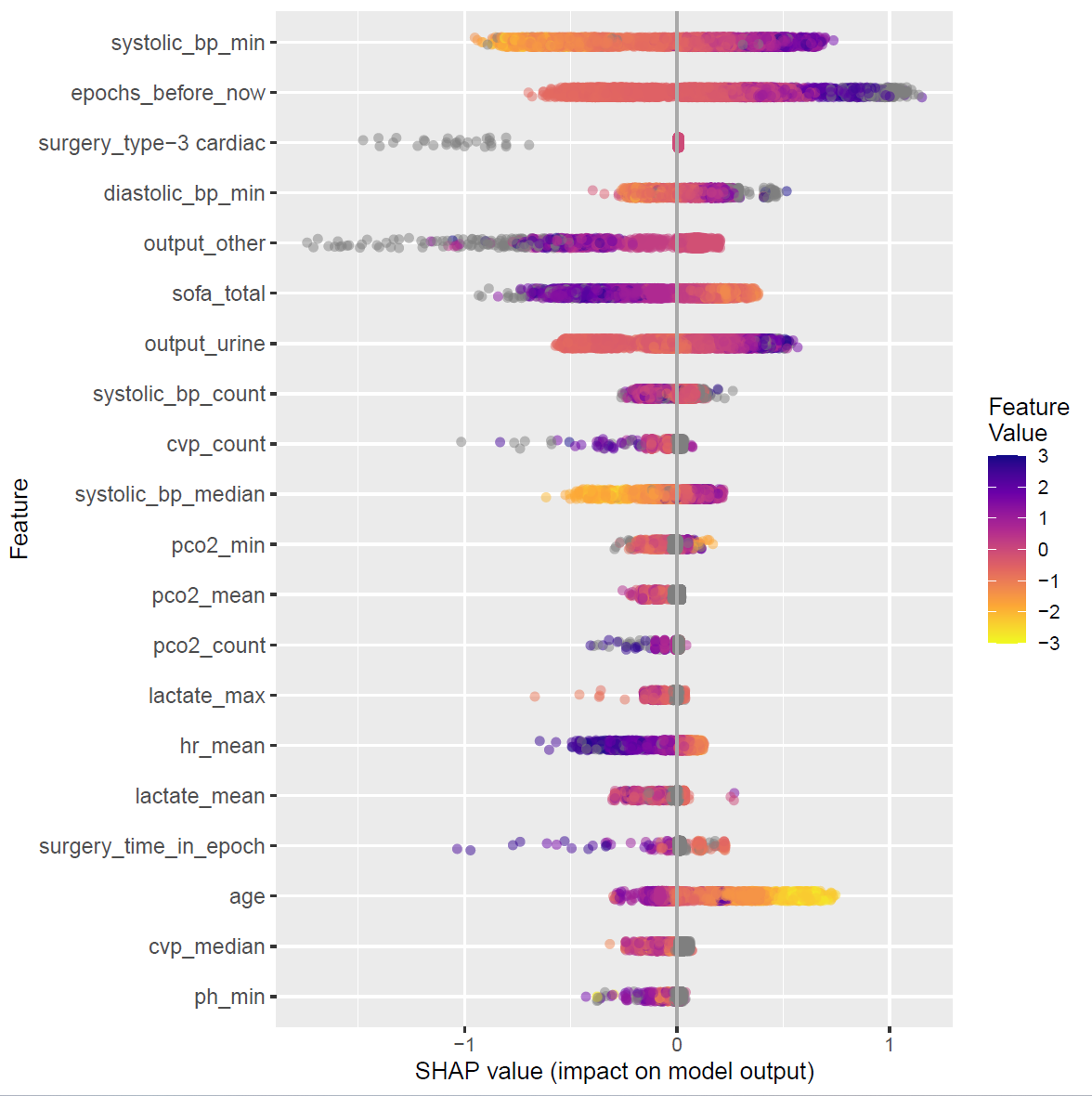

Supplement: 1 [file NIHMS1950985-supplement-1.docx]
